# Recent changes in the epidemiology of *Neisseria meningitidis* serogroup W across the world, current vaccination policy choices and possible future strategies

**DOI:** 10.1080/21645515.2018.1532248

**Published:** 2018-10-26

**Authors:** Robert Booy, Angela Gentile, Michael Nissen, Jane Whelan, Véronique Abitbol

**Affiliations:** a The Discipline of Child and Adolescent Health, Sydney Medical School, University of Sydney, Sydney, New South Wales, Australia; b Westmead Institute of Medical Research, University of Sydney, Sydney, New South Wales, Australia; c Department of Epidemiology, Ricardo Gutiérrez Children’s Hospital, Buenos Aires, Argentina; d Research and Development, GSK Intercontinental, Singapore; e Clinical Research and Development, GSK, Amsterdam, The Netherlands; f Global Medical Affairs, GSK, Rueil Malmaison, France

**Keywords:** *Neisseria meningitidis*, serogroup W, invasive meningococcal disease, vaccination strategies, epidemiology, literature review, case fatality rate

## Abstract

Invasive meningococcal disease (IMD) is a serious disease that is fatal in 5–15% and disabling in 12–20% of cases. The dynamic and unpredictable epidemiology is a particular challenge of IMD prevention. Although vaccination against meningococcal serogroups A (MenA), MenC and, more recently, MenB, are proving successful, other serogroups are emerging as major IMD causes. Recently, surges in MenW incidence occurred in South America, Europe, Australia and parts of sub-Saharan Africa, with hypervirulent strains being associated with severe IMD and higher fatality rates. This review describes global trends in MenW-IMD epidemiology over the last 5–10 years, with emphasis on the response of national/regional health authorities to increased MenW prevalence in impacted areas. Several countries (Argentina, Australia, Chile, the Netherlands and UK) have implemented reactive vaccination campaigns to reduce MenW-IMD, using MenACWY conjugate vaccines. Future vaccination programs should consider the evolving epidemiology of MenW-IMD and the most impacted age groups.

## Introduction

The bacterium *Neisseria meningitidis* only infects humans and causes invasive meningococcal disease (IMD). IMD is a serious disease with case fatality rates of around 10% even with treatment.^1^ It is associated with severe *sequelae* in up to 20% of survivors, including limb amputation, neurological deficits, hearing loss, and other serious disabilities.^2^ The highest incidence of IMD is typically reported in infants <1 year old, with secondary peaks in incidence occurring in adolescents/young adults and sometimes in older adults (≥65 years of age).^3^

Worldwide, most regions have experienced a downward trend in the incidence of IMD in recent years,^4–7^ probably due in part to a combination of active immunization and secular change impacting risk factors for the disease^8,9^ (e.g., the overall improvement of socioeconomic status and changes in attitude towards behavioral risk factors like smoking habits). In the industrialized world, the majority of cases are sporadic (e.g., in the United States [US], less than 2% of cases are associated with outbreaks^10^), but IMD is unpredictable with outbreaks and epidemics characterizing the pathogen since it was first identified.^11,12^

Almost all cases of both endemic and epidemic IMD are caused by one of 6 meningococcal serogroups: A (MenA), MenB, MenC, MenW, MenY, MenX. Their relative importance as causative agents vary considerably across geographical locations, time periods and age of the hosts.^3,13,14^ Frequent unexplained changes in serogroup incidences and emergence of new, more or less virulent strain variants,^3^ and higher CFRs have been observed for specific serogroups^15^ and clonal complexes globally. Meningococci often undergo horizontal gene transfer and spontaneous chromosomal mutations, which cause wide genetic and antigenic diversity between strains.^3,16,17^ This is perhaps best illustrated by unexpected surges in the prevalence of meningococcal serogroups (e.g., for MenY in Scandinavian countries, between 2010 and 2012^18^).

MenW was previously responsible for <5% of IMD cases,^19^ until the first confirmed MenW-caused outbreak in Hajj pilgrims, during 2000.^20^ Since then, this serogroup has become a dominant cause of IMD, albeit so far in a limited number of countries, particularly in Europe, South America, Australia and some parts of Sub-Saharan Africa.^9,14^

In this paper, an overview of the epidemiology of MenW during the last 5–10 years is provided, as well as of the preventive actions taken to date in countries that have experienced an increase of IMD incidence due to MenW.

**Figure 1.**
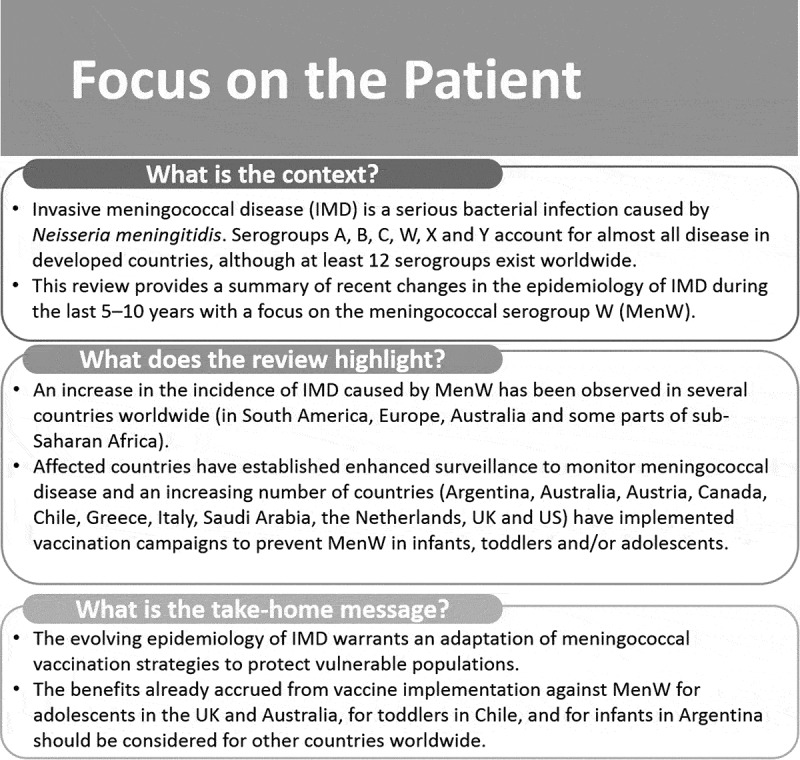
Focus on the patient section.

Figure 1 summarizes the research, clinical relevance and impact on the patient population.

## Global impact of the evolving epidemiology of MenW

MenW was first observed among military personnel in the US in the late 1960s by researchers at the Walter Reed Army Institute of Research,^21^ where the W-135 designation originated. At the proposal of Harrison et al in 2013, the numeric component has since been dropped and the serogroup was renamed simply “W”.^22^ Until the year 2000, MenW was responsible for relatively few cases of IMD and was not reported to cause large outbreaks.^3,23^

A pivotal event was the large outbreak among Hajj pilgrims in Mecca, Saudi Arabia, in the year 2000,^24^ with more than 400 cases and 52 confirmed deaths among the pilgrims and their contacts.^23^ The outbreak was caused by a clone in the sequence type-11 clonal complex (cc11),^24^ and infections with MenW:c11 strains were subsequently reported throughout Europe^24,25^ and the rest of the world,^26–28^ with epidemics in Africa from 2000. Several more outbreaks have since been reported in other countries, including France and the United Kingdom (UK).^23,25^ Over the last 2 decades, the most concerning MenW isolates are those from outbreaks occurring in England and Wales^29^ and the Southern Cone in Latin America^30^; they were shown to belong to the same lineage (the South American/UK cc11 lineage) and were recently proved to be divergent from the Hajj outbreak strain.^31^

As with other meningococci belonging to cc11,^31^ MenW strains causing IMD in recent years are particularly virulent with higher morbidity and CFRs, atypical clinical features, and generally affecting more older adults compared to other common serogroups (A, B, C, X; except maybe Y).^23^ To date, atypical clinical presentations have included septic arthritis and severe respiratory tract infections (including pneumonia, epiglottitis and supraglottitis).^32^ Fatal cases have occurred among adolescents and young adults with MenW septicemia, who presented primarily with gastrointestinal symptoms and then had rapid progression of the disease.^33,34^ In the UK, the CFR observed after the clinical follow-up of all MenW cases (2010–2011 and 2012–2013) was 12%,^29^ similar to that observed during MenC disease surveillance in 1999,^35^ whereas in South America, isolates belonging to this lineage were reported to be even more deadly, with a CFR of 27% estimated during 2012.^36^

**Figure 2.**
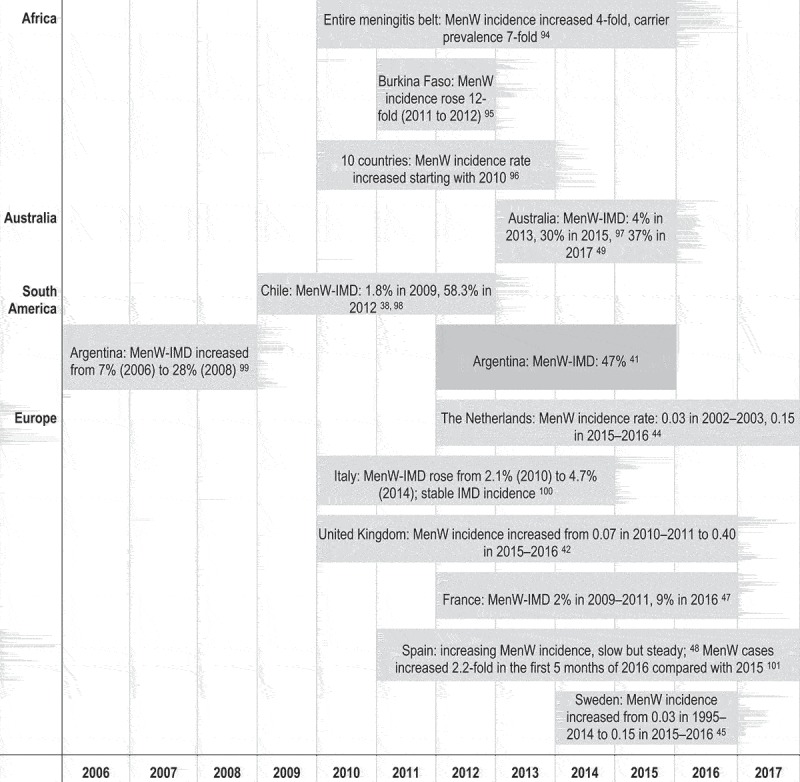
Chronological overview of changes in MenW epidemiology in the last decade (up to July 2018). IMD, invasive meningococcal disease. Note: MenW incidence rate is expressed per 100,000 population.

The chronology of substantial change in the epidemiology of MenW IMD is presented in Figure 2 and an overview of selected relevant papers reporting on MenW IMD incidence is given in **Supplementary Table S1**.

In South America, a localized increase in the prevalence of MenW was first observed in Southern Brazil in the years 2003–2005^37^ and large increases have since been seen in Argentina and Chile.^38,39^ After the first reports in Brazil, Argentina experienced an increase in MenW cases from 2008,^40^ with 78% of all MenW strains belonging to the hypervirulent c11 lineage.^39^ Between 2012 and 2015, MenW was found to account for 47% of total IMD cases in all age groups, and for 46% of IMD in infants <9 months of age.^41^ Infants continue to be the most impacted age group in Argentina, and contrary to findings in other countries, no increase in IMD incidence was observed in adolescents or older adults.^41^

In Chile, the overall incidence of IMD was reported to decrease steadily between 2000 and 2012; however, this trend was not reflected by CFRs which showed a steep increase between 2009 and 2012.^38^ This was due to the emergence of MenW strains belonging to the c11 lineage, which replaced MenB as the most common serogroup in Chile and affected mostly young children 0–5 years of age.^30,38^

Other adjacent countries, like Uruguay and Paraguay, also reported increases in the percentage of MenW-caused IMD in 2008 and 2009.^30,40^

In Europe, the UK and the Netherlands have seen up to 10-fold increases of MenW cases over just a few years.^42,43^ A recent study estimated a relative increase in MenW disease of 79% per year in England (from 30 MenW-IMD cases out of 730 total IMD cases in 2011–2012 to 176 out of 724 in 2014–2015) and of 418% in the Netherlands (from ~4 IMD cases in 2014–2015 to 26 MenW-IMD cases out of 114 total cases in 2015–2016).^44^ The study further suggested the existence of a temporal association between the observed upsurges in these 2 countries.^44^ Moreover, the age distribution of MenW cases in 2015–2016 in the Netherlands was similar to that observed in 2011–2013 in England, with most of the affected cases occurring initially in people aged ≥65 years and later on, in children and adolescents 10–19 years old.^32,44^ In 2014–2015, an age shift towards ≤5 year-olds was observed in the UK.^32^ This trend is yet to be observed in the Netherlands. The increase in MenW disease in these 2 countries was due to cc11 strains, with the prevalence of non-cc11 isolates remaining relatively constant over the considered periods of time.^33,45^ While the MenW strains causing IMD in the UK starting from 2009 belonged to the so-called original UK strain of the South American/UK lineage, if one starts from 2013, a new variant was identified (the so-called UK 2013 strain).^29,31^ Isolates belonging to this strain are now responsible for most MenW cases and the steep increase in IMD incidence, including that observed in the Netherlands between 2014 and 2016,^44^ and have displayed an ability to spread rapidly in the population in addition to their increased virulence.

A surge in the incidence of MenW IMD (from 0.02 to 0.2 per 100,000 population) observed between 2014 and 2016 in Sweden was also attributed mainly to isolates of the UK 2013 strain, with the ≥ 60 and 15–24 year age groups being the most impacted.^45^

A steady increase in MenW incidence was also reported recently in Italy from 2012 to 2014, with no cases reported in infants.^46^

In France, MenW was of less concern after the 2000–2003 global Hajj-associated outbreak, until 2016, when the frequency of MenW cases (albeit still the lowest of all serogroups) increased in comparison to previous years, leading to a reported incidence rate of 0.07 MenW cases/100,000 persons.^47^ Similarly to findings in the other European countries, the majority of cases in 2015 and 2016 were attributed to isolates of the UK 2013 strain, and occurred in a large proportion (94%) in individuals ≥15 years of age.^47^

More recently, during 2016 and the first trimester of 2017, an increase in MenW IMD was also evidenced in Spain, where isolates belonging to the South-American/UK lineage (both the original and the UK 2013 strain) were most commonly identified.^48^

In Australia, IMD incidence was following a decreasing trend up to 2013, when it was reported as 0.6/100,000 population.^49^ However, a recent series of increases was observed to 1.1/100,000 in 2016 and 1.6/100,000 individuals in 2017.^49,50^ This was mainly due to a surge in MenW cases, with notifications doubling from 2014 to 2015 (from 17 to 34 cases, respectively) and continuing to rise sharply in 2016 (108 cases) and 2017 (140 cases out of a total of 383 IMD reported cases).^49^ Furthermore, an outbreak of MenW started in July 2017 in Central Australia with associated cases reported in other parts of the Northern Territory, Queensland, South Australia and Western Australia. The ongoing outbreak is impacting mostly young Aboriginal people in remote Central Australian communities.^51^ A recent outbreak of 4 confirmed MenW cases, was reported in Tasmania.^52^ The hypervirulent cc11 strain was identified for most cases of MenW reported between 2012 and 2017, with the majority of isolated being strain type P1.5,2:F1-1:ST11 or close variants, most of which clustered with isolates identified in the UK and South America during recent years.^53^ MenW-caused IMD occurred more frequently in adults ≥45 years of age (in 2016, MenW caused 59% of IMD in ≥65-year-olds).^50^ However, age-specific incidence of MenW IMD has increased in all age groups from 2015 to 2017, except the 15−19 years age-group where state-based vaccination programs have been introduced.^49,50^ While a large proportion of cases continue to occur in individuals aged ≥65 years, the steepest increase in the notification rates was observed in infants from 2015 to 2017.^49^

## National/regional vaccination recommendations for prevention of IMD

The essential desired effects of vaccination against IMD are firstly to protect vaccinees against IMD when exposed to *N. meningitidis* and also to reduce acquisition, carriage and onward transmission of the bacterium, particularly of hyperinvasive clones.^3^ A characteristic which sets IMD apart from other infectious diseases is that for most circulating serogroups, the age group with the highest carriage (adolescents)^54^ is not the one with the highest incidence (infants). Therefore, experiences with routine vaccination against *N. meningitidis* (particularly MenC campaigns) have historically followed one of possible 3 directions, illustrated with examples below: (i) offer direct protection only to high incidence groups, by targeting directly-impacted groups like infants; (ii) aim for indirect protection by vaccinating the carriers (adolescents), thus trying to prevent transmission to other age groups over time; or (iii) implement both of these strategies by vaccinating the high-incidence groups and offering catch-up campaigns.^3,17^ The latter approach may have the clear advantage of aiming to reduce both disease incidence and carriage.

Vaccination against IMD has been very effective in achieving control of the disease, as shown by several successful campaigns with conjugate meningococcal vaccines.^3,14,16^ In 1999, in response to a steep increase in the number of MenC cases, the UK introduced conjugate MenC vaccine into the routine immunization program, first in infants, and subsequently in a single catch up program for everyone aged 12 months–17 years.^35,55^ The MenC vaccination program in the UK has been seen as a model for other countries across the world, with a similar “children plus catch-up” program (from 14 months of age up to 18 years old included) leading to comparable results in the Netherlands in preventing MenC-IMD.^56^ A different approach was represented by the implementation of routine vaccination with MenC-conjugate vaccine in Brazil in 2010, for infants 3 and 5 months of age, with a booster at 12–15 months. This strategy achieved a rapid impact on the incidence of MenC, but only in the vaccinated age groups, with little to no effect observed in groups not targeted by the program.^57^ As a result, Brazil decided to gradually implement MenC vaccination among adolescents 12–13 years of age starting in 2017, and targeting 9–10-year-olds by 2020.^58^

Based on previous experience with conjugate vaccines used in the control of MenC (as illustrated) and subsequently, MenA-IMD in the African meningitis belt,^59^ a mass vaccination campaign or routine immunization against MenW could also have the potential to decrease the incidence of MenW disease in affected areas, through direct protection and potentially also through indirect effects.^42,60^ To date, a growing number of countries worldwide are implementing vaccination programs to control outbreaks or increases over time of MenW-caused IMD, in view of the hypervirulence of currently circulating MenW strains. In addition, MenACWY vaccination – including booster vaccination after primary immunization against MenC – is recommended in several countries to maintain control over MenC-IMD and provide broader protection against other meningococcal serogroups (Table 1). Several other countries (Italy, the UK, the US, Greece, Austria, most of Canada, the Netherlands and regions in Australia) have included quadrivalent meningococcal vaccines for adolescents in their vaccination programs to provide a MenACWY booster in previously MenC primed individuals.^74^

**Table 1.** Introduction of MenACWY vaccination in national immunization schedules.

	Vaccination program against invasive meningococcal disease			
Direct response to increased MenW incidence	Age group	Vaccination schedule	Year of implementation	Ref.
Chile	≥9 months–5 years	1 dose (a second dose was given in infants ≥9 months)	2012–2014	^36,61^
	1 year	1 dose	2014	
Argentina	>3 months	3 doses at 3, 5 and 15 months of age	2017	^62^
	11 years	1 dose		
United Kingdom	14–18 years	1 dose	2015	^34,63^
Australia, nationally	1 year	1 dose	July 2018	^51,64^
Australian Capital Territory, Victoria, Western Australia, Queensland, Tasmania	15–19 years	1 dose	2017	
	16–18 years	1 dose	2018	
New South Wales	12 months–19 years	1 or 2 doses, depending on age at vaccination and administered vaccine	November 2017	
Northern Territory				
The Netherlands	14 months	1 dose	2018	^65^
	12–14 years	1 dose		
Prevention and control of all-cause meningococcal disease				
Austria	10–13 years	1 dose	2012	^66,67^
Canada*	12–24 years	1 dose	2009	^68,69^
Greece	11–12 years	1 dose	2011	^70,71^
Italy	12–18 years	1 dose	2017	^72^
Saudi Arabia	<2 years	2 doses, 3 months apart	2013	^73^
	2–5-years	1 dose		
United States	11–12 years	1 dose + 1 booster dose at 16 years	2013	^8^

MenACWY, meningococcal quadrivalent conjugate vaccine.

*All provinces and territories, except Manitoba and Quebec.

## Country-specific responses to the changing epidemiology of MenW

### Chile

In Chile, vaccination strategies targeted the age group with the highest incidence and risk of meningococcal disease. In 2012, an immunization campaign was initiated, using conjugate quadrivalent vaccines administered to children 9 months–< 5 years old, as 2-dose series for those < 2 years old and 1-dose for 2–4 year-olds.^75^ From January 1, 2014, the single dose MenACWY vaccination was included in the national immunization schedule and became mandatory for all children at 1 year of age.^61^

The proportion of IMD cases due to MenW in all age groups increased from 58% in 2012 to 65% and 75% in 2013 and 2014, and then remained in the 62–67% range from 2015 to 2017.^76^ The overall yearly incidence of IMD remained ≤ 1/100,000 persons, with estimated annual incidences of 0.8/100,000 in 2012–2014, 0.7/100,000 in 2015, 0.6/100,000 in 2016 and 0.3/100,00 up to September 2017.^76,77^ Vaccine coverage of 95.7% was reported in 2016 in infants aged <1 year.^76^ An early impact of vaccination on the incidence of MenW was only seen for the age groups targeted for the vaccination, as indicated by a reduction of MenW cases of 71% reduction from 2011 to 2016 in children aged 1–5 years.^60^ As an illustration of the threat of the new MenW clone in adolescents, the number of IMD cases in this age group increased over the same period, with most due to MenW.^60^ No reduction of cases in unvaccinated age groups (herd effect of vaccination) occurred following mass vaccination of infants.^3,17,60^

### Argentina

In Argentina, an incidence of 0.44–0.75 IMD cases per year was reported, with a high proportion of IMD cases (47%) caused by MenW between 2012 and 2015.^41^ To reduce the incidence and burden of IMD in infants, starting with January 2017, MenACWY vaccination was included in the national immunization plan for infants >3 months of age, according to a 2 + 1 dose schedule (with vaccine doses administered at 3, 5 months of age and a booster dose at 15 months).^62^ In addition, to reduce carriage by adolescents, a single dose of MenACWY vaccine is provided at 11 years of age.^62^ Active surveillance is ongoing to closely monitor the impact of this national immunization program.

### UK

In the UK, the almost 10-fold increase from 2008/09 to 2014/15 in the number of MenW cases (19 and 170 cases, respectively) was initially observed in the elderly (≥ 65 years old) but rapidly expanded across all age groups, particularly in children aged ≤ 4 years and 15–19-year old adolescents.^32^ This increase led to a decision to offer the MenACWY conjugate vaccine to all adolescents aged from 14–18 years from August 2015.^34^ The program set out to offer the vaccine to the entire cohort during 2015–17, with priority given to secondary school graduates in 2015 because of the recognized high risk of IMD for students about to live in dormitories or university residency halls.^78^ Furthermore, the monovalent MenC vaccination at 14 years of age in the existing vaccination program was replaced by MenACWY and various catch-up programs were targeted at specific groups (adolescents and university entrants 18–25 years of age).^34^

After 1 year, a vaccination coverage rate of ~37% had been attained and a reduction of 69% in the number of MenW cases in this cohort was observed, using trend analysis to compare with the predicted number of cases; the absolute impact was lower, with 218 meningococcal infections still reported in individuals of all ages in 2015–2016.^42^ All confirmed MenW cases (6 among ~ 650,000 individuals) reported in the population eligible for vaccination occurred in unvaccinated individuals.^42^ In a school-based vaccination program undertaken in adolescents, targeting 13–15-year-olds born between September 2000 and August 2002, coverage rates of routinely offered MenACWY vaccination amounting to 77–84% have been achieved^79^; the program aimed to provide direct protection to the vaccinated cohort and indirect protection to other unvaccinated age group by reducing MenW-carriage.^80^ In a recent cross-sectional evaluation of MenW carriage in 1st year university students in the UK, MenW carriage increased over a 6-month period in those studied,^81^ while vaccination coverage in the student population increased from 31% to 70% over the same period.^82^ The increase in MenW carriage was due to expansion of the hypervirulent P1.5,2:F1-1:ST-11 UK 2013 strain; these initial data suggested that the MenACWY vaccine did not prevent carriage of the MenW strain in adolescents.^81–83^ The potential for MenACWY to induce herd protection against MenW strains in the wider population in the UK and elsewhere is as yet uncertain and will require further study.

### Australia

In response to the overall increase in MenW cases since 2014, 5 of 6 states have already implemented temporary, free MenACWY vaccination programs for 15–19 year old adolescents (only 17–18-year olds are targeted in New South Wales).^51^ The programs are planned to last until December 2018 in Victoria and Western Australia, April 2018 in Tasmania, and May 2018 in Queensland. Furthermore, following the MenW-outbreak ongoing since July 2017 in Central Australia particularly affecting indigenous Australians, several states are currently implementing time-limited MenACWY vaccination programs for affected communities.^51^ Thus, in certain regions, the initial program was expanded to include 1 dose of a quadrivalent meningococcal vaccine for all children 12–< 24 months of age in the Northern Territory,^84^ children 1–< 5 years in Western Australia,^85^ and for 1–19-year-olds in South Australia.^86^ As an urgent response to the recent MenW cases reported in Tasmania, MenACWY vaccination will be offered to all individuals aged between 6 weeks and 20 years, as of August 2018.^52^ Additionally, as of July 2018, the national immunization program will include a routine, funded, single dose of MenACWY vaccine at 1 year of age.^64^

### The Netherlands

In the Netherlands, the increase in MenW cases^43^ has led to changes in the national immunization schedule; from May 1, 2018 onwards, MenACWY vaccination is replacing MenC vaccination that was recommended at 14 month of age and, will be offered to all children 13–14 years of age from October 2018.^65^

### Other countries

To date, no other country has launched reactive vaccination campaigns against MenW; however, strict surveillance has been widely instituted and possible vaccination strategies are being considered, based on surveillance trends. MenACWY vaccines are currently approved/licensed for use in different ages in several other regions and countries.^16,17,74^ While many countries around the world recommend MenACWY vaccination for different at-risk populations, travelers to endemic countries or in case of outbreaks,^74^ several countries also include them in their national immunization plan, to control IMD caused by four out of the six clinically-relevant meningococcal serogroups worldwide. In Austria, MenACWY vaccination is offered to children 10–13 years of age and as a booster dose to adolescents vaccinated with MenC during infancy^66,67^ since 2012. Since the year 2001 MenACWY has also been offered to adolescents in Greece (11–12 years of age, since 2011),^70,71^ and Italy (12–18 years of age, since 2017),^72^ while in the Saudi Arabia, the vaccine has been introduced in the national vaccination schedule since 2013 (a 2-dose series at 9 and 12 months in infants, 2 doses administered 3 months apart for children ≥6 months to ≤2 years of age and a single dose in 2–5-year-olds).^73^ In the US, MenACWY is included in the routine vaccination program for adolescents since 2013, as a single dose at 11–12 years, followed by a booster dose at age 16.^8^ In Canada, as of 2017, MenC or MenACWY are recommended for the adolescent booster in all provinces and territories, except Manitoba and Quebec.^68^

## Discussion

Considerable increases in the incidence of MenW and/or in the proportion of all IMD cases caused by MenW have, so far, been seen in considerable number of countries in Europe, South America, Australia and some parts of sub-Saharan Africa, predominantly caused by closely similar strains belonging to the hypervirulent cc11 strain. All the affected countries have established surveillance and, until now, 5 of the countries (Chile, UK, Argentina, Australia, and the Netherlands) have implemented reactive vaccination campaigns directly addressing the increase in MenW-IMD incidence. Other countries have also introduced quadrivalent conjugate vaccines in their immunization program to protect against all-cause meningococcal disease. Surveillance is key to the control of MenW and IMD incidence, and active systems like those in place in Northern America,^87^ the UK^88^ or the Netherlands^44^ are more effective, allowing rapid mapping of emerging disease trends and fast disease control response measures.^13^ Moreover, for all meningococcal infections, in addition to notification of clinical cases using clear and uniform case definitions, surveillance should include laboratory reporting^89^; this is particularly relevant for MenW, in view of the distinct clinical manifestations of the circulating hypervirulent strains. The Global Meningococcal Initiative recently advocated for ongoing surveillance and expansion of vaccination programs, together with recommendations to enable DNA analysis methods (like multi-locus sequence typing and whole-genome sequencing) to further characterize isolates causing IMD.^3^

Determining optimal vaccination strategies is complicated, in particular in countries with relatively low incidence of the disease. One important factor impacting the choice of a vaccination strategy is that carriage rates and disease incidence are both age-related but peaking at differing ages. In a meta-analysis of carriage data from countries where MenB and MenC are predominant, overall meningococcal carriage prevalence was shown to reach its highest at 23.7% in 19 year-olds versus 4.5% in infants and 7.8% in 50 year-olds^54^ whereas IMD incidence peaks in infancy and young children.^17^ Although the impact of MenW carriage on MenW-caused incidence is yet to be elucidated, data from Chile for the year 2013 indicated that carriage of MenW hypervirulent strains among unvaccinated children and adolescents 9–19 years of age is low, and is not correlated with the increase in the incidence of IMD caused by the MenW strain.^90^ In the UK, early data in vaccinated adolescents also suggested no impact of vaccination on MenW carriage.^83^

In Chile, a strategy of aiming the vaccination campaign at the most susceptible age groups to be infected by MenW was effective in reducing the incidence and mortality in the targeted age groups. In Argentina, a strategy targeting both infants and adolescents was implemented, but an assessment of the effects of this combination strategy is not yet available as the vaccination campaign started in 2017. In the UK, the strategy selected was to target the vaccination campaign at the age groups with the highest carrier rates, considered to be the principal responsible for onward transmission. A rapid reduction in the number of MenW cases of 69% as compared to the number predicted by trend analysis was observed in the vaccinated age group^42^; however, increase in MenW carriage in university students has been measured despite high level of vaccine coverage. Further research is required to determine if there is potential for population-wide herd protection.^82^

The possibility that a MenB protein vaccine may also afford some protection against MenW has been discussed.^91^ The MenW:cc11 strain causing IMD in the UK possesses alleles of *Neisseria* adhesin A (NadA) 2/3 peptide variants and neisserial heparin binding antigen (NHBA) peptide 29. NadA and NHBA are 2 of the 4 components of the 4CMenB vaccine licensed for routine immunizations in the UK and therefore the vaccine could potentially induce cross-protection,^91^ especially in case of NadA, for which the variant contained in 4CMenB vaccine was already predicted to be highly cross-reactive with the MenW NadA variant.^92^

Offering one dose of MenACWY conjugate vaccine as a booster to individuals primed with MenC vaccine could have the advantage of maintaining herd protection against MenC, while also providing broader protection against other meningococcal serogroups.^17^ This strategy has already been initiated by the UK, Italy, Greece, Austria,^93^ the US^8^ and Canada^68^ in reaction to the steep increase of IMD incidence.

## Conclusion

The increased incidence and spread of MenW in different parts of the world over approximately the last decade warrants accurate surveillance and prompt action from national health authorities. Positive evidence supporting the effectiveness of MenACWY vaccination campaigns in the control of MenW-IMD in different age groups is emerging from a number of countries worldwide. Whether high levels of vaccination coverage among adolescents, typically carriers of *N. meningitidis*, will result in herd protection is still unconfirmed. However, due to the rapidly-evolving epidemiology of MenW globally, the diversity and virulence of MenW strain variants and the difference in impacted age groups, a responsive vaccination strategy that is tailored to local and regional epidemiology and available resources, will most likely be required.

## Disclosure of potential conflicts of interest

RB’s institution received grants from all vaccine companies operating in Australia for the conduct of several clinical trials, for speaker’s honoraria, for participation in advisory boards and for support of the travel costs linked to conferences and meetings attendances. MN, JW, and VA are employees of the GSK group of companies. MN, JW and VA hold shares in the GSK group of companies as part of their employee remuneration. AG has nothing to disclose.

## Trademark

*Bexsero* and *Menveo* are trademarks owned by the GSK group of companies.
