# Supplementary material for: Recent changes in the epidemiology of Neisseria meningitidis serogroup W across the world, current vaccination policy choices and possible future strategies
Source: Hum Vaccin Immunother. 2018 Oct 26;15(2):470–80. doi: 10.1080/21645515.2018.1532248 (PMC6505668; doi:10.1080/21645515.2018.1532248)
Supplement: Supplemental Material [file khvi-15-02-1532248-s001.docx]

**Table S1.** Selected referenced describing the worldwide epidemiology of invasive meningococcal disease caused by MenW from 2007 to 2017

| Author (publication year) | Country | Epidemiological periods covered | Key incidence findings | Ref. |
| --- | --- | --- | --- | --- |
| *sub-Saharan Africa (meningitis belt)* | | | | |
| Koutangni (2015) | Entire region | 1969–2010 | For MenW, a 4-fold increase in incidence and 7-fold increase in carrier prevalence from endemic (wet season) to hyperendemic (dry season) seasons | [1](#_ENREF_1) |
| Collard (2013) | Niger | 2008–2011 | In 2010, 26% of IMD cases were A, 72% were MenW ST-11  No cases of A in 2011 | [2](#_ENREF_2) |
| Savadogo (2014) | Burkina Faso | 2012 | Outbreak in 2012, MenW identified in 68% of isolates | [3](#_ENREF_3) |
| WHO (2013) | 4 countries | 2012 | Epidemic threshold crossed in Benin, Burkina Faso, Ghana and Chad  MenW caused 52% of case isolates, ST-11 predominant | [4](#_ENREF_4) |
| Nuoh (2016) | Ghana | 2009–2013 | Decreasing trend in IMD incidence, MenY/W made up 40% of IMD | [5](#_ENREF_5) |
| Lingani (2015) | 10 countries | 2004–2013 | Incidence rate of MenW increased from 0.05 to >0.15 after introduction of MenA vaccine | [6](#_ENREF_6) |
| MacNeil (2014) | Burkina Faso | 2012 | 62% of confirmed IMD cases were MenW; in 2012, MenW incidence rate was 8.7in 2012 and 0.7in 2011 | [7](#_ENREF_7) |
| *South America* | | | | |
| Efron (2009) | Argentina | 2006–2008 | MenW as a proportion of IMD cases was 7% in 2006, 13% in 2007, 28% in 2008.  All tested MenW strains were ST-11 | [8](#_ENREF_8) |
| national data | Argentina | 2012–2015 | IMD incidence rate decreased from 2012 (0.75) to 2015 (0.44); MenW accounted for 47% of cases | [9](#_ENREF_9) |
| Sorhouet-Pereira (2013) | Argentina | 2010 | The proportion of MenW-caused IMD cases was 49%; 78% of these were ST-11 | [10](#_ENREF_10) |
| Araya (2014) | Chile | 2006–2012 | In 2011, a rise in IMD incidence due to increase in MenW; in 2012, MenW caused 58% of cases | [11](#_ENREF_11) |
| Araya (2015) | Chile | 2000–2012 | MenW caused 1.8% of all confirmed IMD cases in 2009, 58% in 2012; most MenW were ST-11 | [12](#_ENREF_12) |
| *North America* | | | | |
| Harrison (2010) | United States | 2007 | MenW caused 9% of IMD cases | [13](#_ENREF_13) |
| Doyle (2010) | Florida | 2008–2009 | Cluster of 14 MenW cases in Southeast Florida | [14](#_ENREF_14) |
| *Australia, New Zealand* | | | | |
| Gunaratnam (2013) | Australia | 2003–2012 | MenW caused 14% of cases in elderly (≥65), 4% in those younger; the overall proportion of IMD in elderly increased significantly, from 4% to 6% | [15](#_ENREF_15) |
| Carville (2016) | Australia | 2003–2015 | MenW proportion of IMD was 4% in 2013, 30% in 2015; 9/22 MenW cases in people aged >70 years; 16/22 were W ST-11 | [16](#_ENREF_16) |
| Martin (2016) | Australia | 2013–2015 | Increase in MenW cases as a proportion of IMD from 8% in 2013 to 19% in 2015. Case fatality for MenW was 10.7% and for all IMD combined 4.7% | [17](#_ENREF_17) |
| Lahra (2016) | Australia | 2015–2016 | MenW proportion of IMD in 2015/Q3 was 25%, in 2016/Q3 50% and all IMD had grown by 16% year-on-year | [18](#_ENREF_18), [19](#_ENREF_19) |
| Lopez (2014) | New Zealand | 2008–2013 | MenW proportion of IMD was 9% in 2013, number of MenW cases increased from 2009 to 2010 and then decreased in 2012 | [20](#_ENREF_20) |
| *Asia* | | | | |
| Fukusumi (2016) | Japan | 1999–2014 | MenW caused 3% of IMD; IMD incidence rate was 0.028/100,000 and case fatality ratio was 19% | [21](#_ENREF_21) |
| Wilder-Smith (2010) | Singapore | 2000–2008 | 14 cases of MenW from 2000 to 2003, none since 2004 | [22](#_ENREF_22) |
| Zhou (2013) | China | 2011–2012 | 11 MenW cases identified in Southeast China, all ST-11 | [23](#_ENREF_23) |
| *Europe* | | | | |
| Hill (2015) | United Kingdom | 2010–2012 | MenW caused 6% of IMD cases | [24](#_ENREF_24) |
| Ladhani (2015) | England and Wales | 2010–2013 | Age distribution of all MenW cases; MenW as proportion of IMD increased each year from 2009 from the historical 1-2% | [25](#_ENREF_25) |
| Oldfield (2017) | United Kingdom | 2015–2016 | Carriage among first-year students was 0.7% in Sep 15, 8% in March 2016 | [26](#_ENREF_26) |
| Campbell (2015) | United Kingdom | 2008–2015 | MenW proportion of IMD was 1.7% in 2008/9, 7% in 12/13, 15% in 13/14, 25% in 14/15  For >65 year-olds, the W proportion was 49%, 32% for 15-19 y, 15% for < 1 year | [27](#_ENREF_27) |
| Campbell (2017) | England | 2010-16 | Overall MenW incidence rates increased from 0.07 in 2010-11 to 0.17 in 2013-14, 0.35 in 2014-15 and 0.40 in 2015-16 | [28](#_ENREF_28) |
| Russcher (2017) | Netherlands | 2002–2016 | MenW incidence rate was 0.03 in 2002-14, 0.29 in 2016; unusual and severe presentation common for MenW | [29](#_ENREF_29) |
| Knol (2017) | Netherlands | 2005–2017 | MenW incidence was 0.29 in 2016 and increased to 0.57 in 2017 (extrapolation from observations up to March 2017) | [30](#_ENREF_30) |
| ECDC (2012) | European Economic Area | 2008–2012 | Overall decrease of IMD incidence, only MenY incidence has been increasing in some places | [31](#_ENREF_31) |
| Stefanelli (2015) | Italy | 2010–2014 | Increase in MenW as a proportion of all IMD cases from 2.1% to 4.7%. | [32](#_ENREF_32) |
| Hong (2017) | France | 2000–2016 | IMD caused by MenW emerged in 2000, then declined but increased again from 2012. No precise epi-data given, but shift in the age distribution to older ages. Increase due to the UK-South America strain | [33](#_ENREF_33) |
| Abad (2017) | Spain | 2016–2017 | Increasing incidence of MenW, characterized as “a slow spread of MenW ST-11 clonal complex strains associated with the South-American sublineage” perhaps starting in 2011 | [34](#_ENREF_34) |

MenW, meningococcal serogroup W; IMD, invasive meningococcal disease.

Note: MenW incidence rate is expressed per 100,000 population

**References:**

1. Koutangni T, Boubacar Mainassara H, Mueller JE. Incidence, carriage and case-carrier ratios for meningococcal meningitis in the African meningitis belt: a systematic review and meta-analysis. PLoS One. 2015;10(2):e0116725. doi: 10.1371/journal.pone.0116725.

2. Collard JM, Issaka B, Zaneidou M, Hugonnet S, Nicolas P, Taha MK, Greenwood B, Jusot JF. Epidemiological changes in meningococcal meningitis in Niger from 2008 to 2011 and the impact of vaccination. BMC Infect Dis. 2013;13:576. doi: 10.1186/1471-2334-13-576.

3. Savadogo M, Kyelem N, Yelbeogo D, Koussoube D, Tarbagdo F, Ouedraogo A. [The *Neisseria meningitidis* W135 epidemic in 2012 in Burkina Faso]. Bull Soc Pathol Exot. 2014;107(1):15-7. doi: 10.1007/s13149-013-0320-y.

4. Meningococcal disease in countries of the African meningitis belt, 2012 - emerging needs and future perspectives. Wkly Epidemiol Rec. 2013;88(12):129-36.

5. Nuoh RD, Nyarko KM, Nortey P, Sackey SO, Lwanga NC, Ameme DK, Nuolabong C, Abdulai M, Wurapa F, Afari E. Review of meningitis surveillance data, upper West Region, Ghana 2009-2013. Pan Afr Med J. 2016;25(Suppl 1):9. doi: 10.11604/pamj.supp.2016.25.1.6180.

6. Lingani C, Bergeron-Caron C, Stuart JM, Fernandez K, Djingarey MH, Ronveaux O, Schnitzler JC, Perea WA. Meningococcal meningitis surveillance in the African Meningitis Belt, 2004-2013. Clin Infect Dis. 2015;61 Suppl 5:S410-5. doi: 10.1093/cid/civ597.

7. MacNeil JR, Medah I, Koussoube D, Novak RT, Cohn AC, Diomande FV, Yelbeogo D, Kambou JL, Tarbangdo TF, Ouedraogo-Traore R, et al. *Neisseria meningitidis* serogroup W, Burkina Faso, 2012. Emerg Infect Dis. 2014;20(3):394-9. doi: 10.3201/eid2003.131407.

8. Efron AM, Sorhouet C, Salcedo C, Abad R, Regueira M, Vazquez JA. W135 invasive meningococcal strains spreading in South America: significant increase in incidence rate in Argentina. J Clin Microbiol. 2009;47(6):1979-80. doi: 10.1128/jcm.02390-08.

9. Ladhani SN, Giuliani MM, Biolchi A, Pizza M, Beebeejaun K, Lucidarme J, Findlow J, Ramsay ME, Borrow R. Effectiveness of meningococcal B vaccine against endemic hypervirulent *Neisseria meningitidis* W strain, England. Emerg Infect Dis. 2016;22(2):309-11. doi: 10.3201/eid2202.150369.

10. Sorhouet-Pereira C, Efron A, Gagetti P, Faccone D, Regueira M, Corso A, Argentinean SIREVA II Working Group, Gabastou JM, Ibarz-Pavon AB. Phenotypic and genotypic characteristics of *Neisseria meningitidis* disease-causing strains in Argentina, 2010. PLoS One. 2013;8(3):e58065. doi: 10.1371/journal.pone.0058065.

11. Araya P, Diaz J, Seoane M, Fernandez J, Terrazas S, Canals A, Vaquero A, Barra G, Hormazabal JC, Pidal P, et al. [Laboratory surveillance for invasive meningococcal disease in Chile, 2006-2012]. Rev Chilena Infectol. 2014;31(4):377-84. doi: 10.4067/s0716-10182014000400001.

12. Araya P, Fernandez J, Del Canto F, Seoane M, Ibarz-Pavon AB, Barra G, Pidal P, Diaz J, Hormazabal JC, Valenzuela MT. *Neisseria meningitidis* ST-11 clonal complex, Chile 2012. Emerg Infect Dis. 2015;21(2):339-41. doi: 10.3201/eid2102.140746.

13. Harrison LH. Epidemiological profile of meningococcal disease in the United States. Clin Infect Dis. 2010;50 Suppl 2:S37-44. doi: 10.1086/648963.

14. Doyle TJ, Mejia-Echeverry A, Fiorella P, Leguen F, Livengood J, Kay R, Hopkins R. Cluster of serogroup W135 meningococci, southeastern Florida, 2008-2009. Emerg Infect Dis. 2010;16(1):113-5. doi: 10.3201/eid1601.091026.

15. Gunaratnam P, Massey P, Durrheim D, Torvaldsen S. Invasive meningococcal disease in elderly people, New South Wales, Australia, 1993 to 2012. Western Pac Surveill Response J. 2013;4(4):4-10. doi: 10.5365/wpsar.2013.4.4.001.

16. Carville KS, Stevens K, Sohail A, Franklin LJ, Bond KA, Brahmi A, Romanes F, Ong KS. Increase in meningococcal serogroup W disease, Victoria, Australia, 2013-2015. Emerg Infect Dis. 2016;22(10):1785-7. doi: 10.3201/eid2210.151935.

17. Martin NV, Ong KS, Howden BP, Lahra MM, Lambert SB, Beard FH, Dowse GK, Saul N, Communicable Diseases Network Australia MenW Working Group. Rise in invasive serogroup W meningococcal disease in Australia 2013-2015. Commun Dis Intell Q Rep. 2016;40(4):E454-E9.

18. Lahra MM, Enriquez RP, National Neisseria Network. Australian Meningococcal Surveillance Programme annual report, 2015. Commun Dis Intell Q Rep. 2016;40(4):E503-E11.

19. Lahra MM, Enriquez RP. Australian Meningococcal Surveillance Programme, 1 July to 30 September 2016. Commun Dis Intell Q Rep. 2016;40(4):E560.

20. Lopez L, Sherwood J. The Epidemiology of Meningococcal Disease in New Zealand in 2013. 2014, Institute of Environmental Science and Research Ltd (ESR) Wellington, New Zealand. Available from https://surv.esr.cri.nz/PDF_surveillance/MeningococcalDisease/2013/2013AnnualRpt.pdf [accessed 10 January 2018].

21. Fukusumi M, Kamiya H, Takahashi H, Kanai M, Hachisu Y, Saitoh T, Ohnishi M, Oishi K, Sunagawa T. National surveillance for meningococcal disease in Japan, 1999-2014. Vaccine. 2016;34(34):4068-71. doi: 10.1016/j.vaccine.2016.06.018.

22. Wilder-Smith A, Chow A, Goh KT. Emergence and disappearance of W135 meningococcal disease. Epidemiol Infect. 2010;138(7):976-8. doi: 10.1017/s095026880999104x.

23. Zhou H, Liu W, Xu L, Deng L, Deng Q, Zhuo J, Shao Z. Spread of *Neisseria meningitidis* serogroup W clone, China. Emerg Infect Dis. 2013;19(9):1496-9. doi: 10.3201/eid1909.130160.

24. Hill DM, Lucidarme J, Gray SJ, Newbold LS, Ure R, Brehony C, Harrison OB, Bray JE, Jolley KA, Bratcher HB, et al. Genomic epidemiology of age-associated meningococcal lineages in national surveillance: an observational cohort study. Lancet Infect Dis. 2015;15(12):1420-8. doi: 10.1016/s1473-3099(15)00267-4.

25. Ladhani SN, Beebeejaun K, Lucidarme J, Campbell H, Gray S, Kaczmarski E, Ramsay ME, Borrow R. Increase in endemic *Neisseria meningitidis* capsular group W sequence type 11 complex associated with severe invasive disease in England and Wales. Clin Infect Dis. 2015;60(4):578-85. doi: 10.1093/cid/ciu881.

26. Oldfield NJ, Cayrou C, AlJannat MAK, Al-Rubaiawi AAA, Green LR, Dada S, Steels OD, Stirrup C, Wanford J, Atwah BAY, et al. Rise in group W meningococcal carriage in university students, United Kingdom. Emerg Infect Dis. 2017;23(6):1009-11. doi: 10.3201/eid2306.161768.

27. Campbell H, Saliba V, Borrow R, Ramsay M, Ladhani SN. Targeted vaccination of teenagers following continued rapid endemic expansion of a single meningococcal group W clone (sequence type 11 clonal complex), United Kingdom 2015. Euro Surveill. 2015;20(28). doi: 10.2807/1560-7917.ES2015.20.28.21188.

28. Campbell H, Edelstein M, Andrews N, Borrow R, Ramsay M, Ladhani S. Emergency meningococcal ACWY vaccination program for teenagers to control group W meningococcal disease, England, 2015-2016. Emerg Infect Dis. 2017;23(7):1184-7. doi: 10.3201/eid2307.170236.

29. Russcher A, Fanoy E, van Olden GDJ, Graafland AD, van der Ende A, Knol MJ. Necrotising fasciitis as atypical presentation of infection with emerging *Neisseria meningitidis* serogroup W (MenW) clonal complex 11, the Netherlands, March 2017. Euro Surveill. 2017;22(23). doi: 10.2807/1560-7917.es.2017.22.23.30549.

30. Knol MJ, Ruijs H, De Melker H, Sanders L, Van der Ende A. Increase in invasive serogroup W meningococcal disease since 2015 in the Netherlands. 14th Congress of the EMGM, European Meningococcal and Haemophilus Disease Society. September 18-21, 2017. Prague, Czech Republic.

31. Borrow R, Abad R, Trotter C, van der Klis FR, Vazquez JA. Effectiveness of meningococcal serogroup C vaccine programmes. Vaccine. 2013;31(41):4477-86. doi: 10.1016/j.vaccine.2013.07.083.

32. Stefanelli P, Fazio C, Neri A, Rezza G, Severoni S, Vacca P, Fasciana T, Bisbano A, Di Bernardo F, Giammanco A. Imported and indigenous cases of invasive meningocococcal sisease W:P1.5,2:F1-1: ST-11 in migrants' reception centers. Italy, June-November 2014. Adv Exp Med Biol. 2016;897:81-3. doi: 10.1007/5584_2015_5006.

33. Hong E, Barret AS, Terrade A, Denizon M, Antona D, Aouiti-Trabelsi M, Deghmane AE, Parent du Chatelet I, Levy-Bruhl D, Taha MK. Clonal replacement and expansion among invasive meningococcal isolates of serogroup W in France. J Infect. 2017;76(2):149-15. doi: 10.1016/j.jinf.2017.10.015.

34. Abad R, Lopez EL, Debbag R, Vazquez JA. Serogroup W meningococcal disease: global spread and current affect on the Southern Cone in Latin America. Epidemiol Infect. 2014;142(12):2461-70. doi: 10.1017/s0950268814001149.
